# Nucleic Acids in Cancer Diagnosis and Therapy

**DOI:** 10.3390/cancers12092597

**Published:** 2020-09-11

**Authors:** Taewan Kim

**Affiliations:** 1 Department of Anatomy, Histology and Developmental Biology, Base for International Science and Technology Cooperation, Carson Cancer Stem Cell Vaccines R&D Center, International Cancer Center, Shenzhen University Health Science Center, Shenzhen 518055, China; kim.2203@buckeyemail.osu.edu or taewankim@szu.edu.cn; 2 The Ohio State University Comprehensive Cancer Center, Columbus, OH 43210, USA

**Keywords:** noncoding RNA, RNA-binding protein, circular RNA, tRNA fragment, cell-free DNA, cell-free RNA, RNA editing and modification, cancer

## Introduction

Cancer research has been focused on the coding genes occupying 1–2% of the human genome [1]. Moreover, cancer research in the coding genes has been concentrated on their genetic mutations and protein activities/functions. On the other hand, noncoding genomic areas and noncoding RNAs have been less weighed, frequently being considered as genetic noise and by-products. Nevertheless, most cancer-related abnormalities, including genetic mutations, are found in the genomic areas outside of coding genes [2].

As the functions of noncoding RNAs in cancer have been revealed, cancer researchers have been expanding their focus from the 1–2% coding genes to the 98–99% noncoding transcripts of the human genome. As a result, a number of noncoding RNAs and their functional mechanisms have been identified and characterized in most types of cancer [3,4]. The modifications of genomic DNA and RNA also provide new insight into the functional mechanisms of nucleic acids in cancer [5,6,7,8]. Various DNA modifications regulate the expression of coding and noncoding genes beyond the traditional gene regulation mechanism through transcription factors [6,7]. Moreover, the RNA modifications modulate the functional activity of coding and noncoding RNAs [5,8]. Therefore, the scientific view of DNA and RNA as generators and by-products of proteins has been changing. According to the nucleic acid-centered view in the post-central dogma world, rather, coding genes and proteins are the accessories of nucleic acids.

Nucleic acids are also examined as biomarkers of cancer. The DNA and RNA fragments referred to as circulating nucleic acids are being found in not only cancer cells but also extracellular environments including the bloodstream and body fluids [9,10,11]. Therefore, some cancer-specific circulating DNA or RNA fragments in cancer patients could be novel markers for cancer diagnosis. Thanks to the convenience and accuracy of nucleic acid detection, the unique nucleic acid markers expressed in cancer and/or circulating in extracellular environments will provide enormous benefits in clinical cancer detection.

Many scientists have started to focus on the function and potential of DNA and RNA as main targets in cancer research. Eventually, the increasing research interest in nucleic acids has increased the research interest in the functions and mechanisms of nucleic acids featuring RNA-binding proteins, DNA/RNA modifications, cell-free circulating DNA/RNA, and unique subtypes of RNA including tRNA fragments and circular RNAs, as well as well-established noncoding RNAs such as microRNAs and long noncoding RNAs.

This Special Issue intends to focus on the potential of nucleic acids, their modifications, and associated proteins. It will hopefully contribute the frame-shift from the protein-(coding gene) centered to the nucleic acid-centered view of cancer research.
